# Reform and practice of the cross-integration teaching model: A case study of visual communication design

**DOI:** 10.1371/journal.pone.0327813

**Published:** 2025-07-11

**Authors:** Jian Wang, Haizhou Liu

**Affiliations:** 1 Nanjing University of the Arts, Nanjing, China; 2 Huzhou Vocational & Technical College, Huzhou, China; De la Salle University, PHILIPPINES

## Abstract

In the context of digitalization, how to effectively carry out interdisciplinary teaching in the field of visual communication design (VCD) to enhance students’ comprehensive innovation ability and interdisciplinary collaboration skills has become one of the key issues in the current educational reform. Based on STEAM education, this study constructed a cross-integration teaching model (CITM) for VCD from four aspects: top-level design of curriculum, innovations in teaching methods and approaches, integration of interdisciplinary teaching teams and industry resources, and optimization of evaluation and feedback mechanism. This study adopted the action research, taking the Brand and User Experience Design course as a case example. Data were collected through classroom observation, interviews, questionnaires and team discussions, and multi-dimensional qualitative analysis was conducted using triangulation. The study found that the CITM effectively improved students’ innovative thinking, interdisciplinary collaboration and practical abilities, and students showed a high level of acceptance of project-based teaching and co-teaching by multiple teachers. However, the study also revealed that some students still faced difficulties in understanding interdisciplinary theory, and the coordination and coherence of teacher team collaboration need to be further enhanced. It is necessary to further optimizing the communication and collaboration mechanisms of teaching team, strengthening the connection of course content, and continuously introducing industry resources to enhance the application of the model in broader educational contexts.

## 1. Introduction

Against the backdrop of informatization and intelligence, the intersection and integration of design and art disciplines are increasingly emerging as new directions for discipline development and talent cultivation. In recent years, STEAM, as an interdisciplinary education that integrates science, technology, engineering, arts, and mathematics, has advocated interdisciplinary integration and emphasized the cultivation of students’ comprehensive innovation ability to adapt to the complex and evolving needs of the digital age [1,2]. As a highly integrated field, the art of design encompasses both the humanities and social sciences as well as the natural sciences, its value lies not only in the mastery of design skills themselves, but more importantly in the ability to solve practical problems with innovative thinking and practical competence from an interdisciplinary perspective [3].

With the rapid development of digital technologies, the concept and application of VCD in the era of omni-media have far surpassed those of traditional print media and are widely involved in emerging fields such as digital technology, virtual reality (VR), augmented reality (AR), and interaction design [4–8]. The demand for visual design talents in society and industry has undergone fundamental changes. Increasingly, enterprises and institutions require designers not only to possess traditional design skills, but also to demonstrate diversified ways of thinking, interdisciplinary knowledge backgrounds and comprehensive innovative abilities. This change requires design education to keep pace, not only to teach students practical skills, but also to help them develop the abilities to collaborate and innovate across fields in social and industrial environments. Consequently, the cultivation of design talent has shifted from a focus on single skills to an emphasis on comprehensive competence and innovative ability, which prompts higher education institutions are facing new challenges and pressures for educational reform [9]. At present, most VCD majors still adopt the model of monodisciplinary teaching. The curriculum tends to be relatively fixed and lacks flexibility, resulting in insufficient interdisciplinary interaction, which restricts the development of students’ interdisciplinary collaboration ability [10,11]. Specifically, VCD education in Chinese universities currently faces several key issues: First, there is a disconnect between course content and the needs of industry. Second, the mechanisms for interdisciplinary integration and collaboration are inadequate. Third, faculty members often have homogeneous professional backgrounds and limited experience in teamwork. These problems not only constrain students’ comprehensive innovative capabilities but also reduce their competitiveness in the job market. Therefore, in response to these issues, this study aims to construct the CITM for VCD that is suited to the digital era.

Therefore, the core issue of this study was: How to construct and implement the CITM based on the STEAM education to enhance the interdisciplinary collaboration and comprehensive innovation abilities of students majoring in VCD? In response to this question, the purposes of this study were: (1) to design a modular interdisciplinary curriculum system that promotes the integration and practical application of interdisciplinary knowledge; (2) to explore and implement innovative teaching strategies, such as project-based learning (PBL), teamwork and interdisciplinary workshop, in order to promote in-depth integration within the design discipline and between design and other disciplines; (3) to integrate interdisciplinary teaching teams and industry resources to enhance the industry relevance of teaching content; and (4) to evaluate the effectiveness and applicability of the CITM through action research and multi-dimensional evaluation methods, further using triangulation to cross-validate findings from different perspectives, thereby improving the objectivity and reliability of research conclusions.

## 2. Literature review

### 2.1. Interdisciplinary education

With the continuous innovation of global education models, the concept of interdisciplinary education has gradually become an important direction of education reform in the 21st century. STEAM education can effectively cultivate students’ creativity and design thinking [12,13]. Its core lies in promoting students’ creativity and practical problem solving through interdisciplinary collaboration. In particular, it emphasizes the integration of arts with science and technology, highlighting the critical role of art in cultivating creative thinking [14]. Studies have shown that STEAM education has a significant impact on cultivating students’ critical thinking, innovation, and collaboration skills [15–17]. For example, “Maker Movement” integrates science, technology and art through hands-on experiments and PBL to stimulate students’ interest in active learning [18]. Costantino proposed a creative inquiry process framework based on STEAM curricula, an iterative framework that integrates pedagogies of art and design [19], illustrated by practices such as showcasing ideas in exhibitions [20].

STEAM education can provide a new perspective and method for teaching VCD. First, the STEAM model emphasizes collaborative innovation between disciplines, breaking the barriers of traditional single-subject teaching, which is of great significance for cultivating interdisciplinary thinking of students majoring in VCD. In VCD teaching, integrating technology and engineering knowledge, such as digital technology [4], interaction design and VR, can spark students’ creativity and enhance their ability to apply technology, thereby strengthening their capacity to address complex design requirements. Secondly, STEAM education advocates a problem-based learning approach, which aligns well with the project-driven teaching model in VCD. By working with authentic problem scenarios in design practice, students can comprehensively apply scientific knowledge and artistic expression as they explore and solve problems, thereby enhancing their critical thinking and innovation skills. Furthermore, STEAM education model emphasizes student initiative, encourages teamwork, and promotes open-minded thinking, which are equally crucial in VCD. Through team-based and interdisciplinary collaboration, students can interpret design problems from multiple perspectives, resulting in visual expressions with greater depth and impact. Finally, the artistic dimension within STEAM model is particularly crucial, as it infuses VCD with aesthetic and humanistic inspiration. Art, serving as a bridge between technology and society, not only imparts aesthetic value to visual design works but also endows them with cultural significance and social influence. Therefore, integrating STEAM education into VCD teaching helps develop students’ comprehensive abilities, enabling them to cultivate innovative design skills and a sense of social responsibility, thus preparing them to meet diverse design challenges in the future.

Apart from STEAM, some other theories also play an important role in interdisciplinary education. Constructivist theory emphasizes the learner’s active participation in the process of knowledge construction, building on existing experiences. Through interdisciplinary projects and real problem situations, the integration and application of knowledge can be achieved more effectively [21]. For example, Niederriter et al. developed an educational program for students in the healthcare fields using the Constructivist/Active Learning theoretical framework, based on interprofessional simulation. The results showed that students reported high levels of satisfaction with the education, and their interprofessional competencies were also improved [22]. Moreover, the experiential learning theory points out that knowledge is constructed through the reflection and reconstruction of practical experience, and this process has a natural fit with the practice-oriented and PBL emphasized in interdisciplinary education [23]. Specifically, the interdisciplinary teaching in VCD can provide students a wealth of practical experiences, such as PBL and interdisciplinary workshop, while also incorporating emerging technologies like VR and AR to promote students to facilitate in-depth learning experiences and reflection processes, thereby enhancing creativity and problem-solving abilities.

Multiple literature reviews indicated that there is a substantial body of research on interdisciplinary education. In particular, studies on the CITM exhibited diverse perspectives and broad horizons [24–26]. By comparison, Chinese scholars primarily focused on theoretical discussions or literature reviews, whereas foreign scholars were more inclined toward empirical analyses based on specific case studies [25]. In recent years, domestic studies have gradually drawn on international case analysis methods, and some enlightening conclusions have been drawn through comparative studies and literature reviews [27]. Most of these studies have remained at the stage of formal imitation and superficial claims, lacking in-depth systematic research and practical explorations tailored to China’s national conditions. Therefore, current domestic studies in the field of interdisciplinary education still lack systematic quantitative research, particularly sample-based investigations into the practice of interdisciplinary education across different disciplines or professional fields in universities. By collecting first-hand data from a holistic perspective and systematic framework of interdisciplinary and professional integration, and then further organizing and analyzing this data, it is possible to build effective mechanisms to promote the cultivation of VCD talent. In doing so, establishing interdisciplinary strategies and teaching systems for curriculum development can help advance the reform of the CITM. The characteristics of VCD education should be diverse, organized, and intersectional, aiming to embody the spirit of variety, adaptability, and differentiation [10]. This means that during the teaching process, educators should employ diverse instructional methods and a variety of content to stimulate students’ interest in learning, while fostering a willingness to think critically, keen observational skills, and adaptability.

The CITM is based on the demands for diversity, specialization, and organization, as well as the prevailing trends in social development, ultimately exhibiting a complex state of intersection and integration. According to the American scholar Klein, “once a university has organized its activities around realities or problems, not only the disciplines but also the interdisciplinarity is no longer a pedagogical approach or a vision, but an organizational need [28].” Within the teaching curriculum, priority is given to project-driven practical instruction, marking a shift from the traditional knowledge-transmission model to one centered on cultivating innovative practical capability. By embracing the CITM, this transformation promotes synergy, fusion, and mutual complementarity between VCD and other fields or disciplines. Gardner pointed out that creativity often arises at the intersection of disciplines, and the cross-integration of diverse skills can drive innovation [29]. Consequently, interdisciplinary synergy and cross-professional collaboration have progressively become defining features of knowledge innovation. In this context, the knowledge system of VCD needs to have cross-disciplinarity, heterogeneity and comprehensiveness.

### 2.2. Curriculum reform and practice in VCD education

In recent years, with the rapid development of the design industry and the continuous innovation of technology, VCD education has gradually realized that the traditional teaching mode is difficult to meet the needs of the interdisciplinary and complicated industry, and curriculum reform has become one of the important topics in major universities and academic research. The research on the reform of VCD education has encompassed a variety of teaching techniques and methods. Digital technology, emerging as a new form of artistic expression, has propelled VCD into a new stage of development. As digital technology continues to mature, the digital expression of VCD has become a prominent trend within the field [4]. Therefore, colleges have placed increasing emphasis on integrating digital skill cultivation into curriculum reforms, closely aligning the latest technological tools with teaching content. Based on digital technology, Bian & Ji systematically studied the teaching of VCD from the aspects of application of design thinking and construction of design methods [4]. In addition, some scholars have investigated how to enhance the teaching effect of VCD through emerging technologies such as big data, VR and AR. Big data technology can help the teaching of VCD to fully excavate the image, text, shape and other information of artworks [30]. For example, Yuan & Wang conducted a study on the teaching practice of VCD in universities based on the deep learning model of big data technology, and their findings indicated a significant improvement in student learning outcomes [31]. Some studies have shown that using VR technology in the teaching of VCD can help students improve their thinking ability and generate new ideas [32]. For example, employing VR technology to manipulate visualizations yields better visual effects than traditional graphic design techniques [33]. Jiawei & Mokmin discussed the development trend of immersive learning using VR technology in VCD education through a systematic literature review [5]. Technology-driven curriculum reform not only expands students’ technical ability, but also promotes their understanding and adaptation to future trends in design.

Traditional VCD teaching is predominantly teacher-centered, with a one-way flow of information in the classroom, which limits students’ participation and creativity. In the curriculum reform, the innovation of teaching methods has become a key focus of practice, primarily reflected in the diversified teaching approaches and the student-oriented educational philosophy. Highly interactive teaching methods such as the flipped classroom [34], group collaboration [35] and design workshop [36] have been widely adopted, addressing the limitations of traditional classroom teaching. Some theories from other disciplines have also been applied to the teaching of VCD. For example, Yang & Hsu validated the feasibility of applying narrative theory to visual design courses through practical teaching activities, and found that the application of narrative techniques to VCD not only facilitates the creativity of designers, but also elicits the audience’s visual memory, thereby encouraging a bidirectional communication between the two entities [37]. Other studies have focused on analyzing specific teaching cases, such as the project-based teaching in brand design courses or creative thinking training in poster design [38,39]. These studies provide crucial technical support and practical references for the reform of art and design curriculum. In summary, although substantial practical achievements have been made in the curriculum reform of VCD, the existing research and practice are largely confined to certain specialized areas and lack the systematic construction and verification of interdisciplinary course model. Furthermore, there has been limited exploration into the design of interdisciplinary curriculum systems, and a comprehensive framework integrating both theory and practice has yet to be established.

## 3. Construction of the CITM

**Fig 1:**
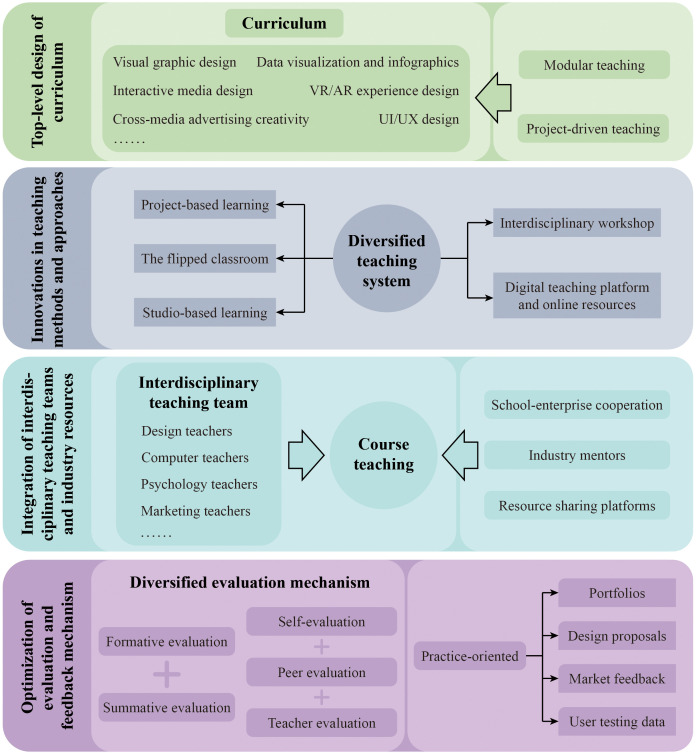
The cross-integration teaching model and implementation path.

This study constructed the CITM and implementation path based on STEAM, focusing on the top-level design of curriculum system, innovations in teaching methods and approaches, the integration of faculty and industrial resources, and the optimization of evaluation and feedback mechanism, as shown in Fig 1. Through the integration of both internal and external faculty resources, as well as extensive industrial resources, the curriculum system establishes interdisciplinary teaching teams and industry-academia collaboration platforms. Combining practice-driven teaching methods with diversified evaluation mechanisms, the curriculum enhances students’ overall competencies by uniting theory and practice, thereby providing both theoretical foundation and practical path for the innovation and development of interdisciplinary design education.

### 3.1. Top-level design of curriculum

The construction of the CITM aims to cultivate students’ comprehensive innovative capabilities and cross-domain collaboration skills through disciplinary intersections and the integration of knowledge. The top-level design of curriculum system, as the key link of constructing this model, emphasizes the integration of multidisciplinary knowledge and the modular design of curriculum structure, so as to meet the needs of the future society for inter-disciplinary talent. The curriculum design integrates core knowledge from fields such as VCD basic courses, digital media technology, user experience research, brand planning, marketing, and social and cultural studies, to meet the comprehensive development needs of students in design expression, technical application, business understanding, and social adaptability. At the same time, the curriculum design adheres to the principles of interdisciplinary collaboration, practice-driven learning, and modular flexibility, emphasizing the integration of theory and practice to ensure that the curriculum system is both cutting-edge and highly applicable.

In terms of the integration of specific course content, the curriculum system is developed from three levels: foundation, core and application, and cross-professional course groups are set up to realize the cross-domain expansion of students’ knowledge structure. The curriculum group includes modules such as visual graphic design, interactive media design, cross-media advertising creativity, UI/UX design, data visualization and infographics, and VR/AR experience design. Visual graphic design, as a basic course in VCD, provides students with the abilities to use design language and aesthetic expression [40]. Interactive media design and UI/UX design focus on user experience and interaction technology, focusing on students’ design ability in digital and information scenarios [41]. The courses of cross-media advertising creativity and data visualization cultivate students’ abilities to integrate brand planning and data analysis to enhance the commercial value and communication effect of design [42]. VR/AR experience design provide students with opportunities to explore future technology applications [5,43]. Through the setting of these modules, the curriculum system realizes the deep integration of art, technology and business. In addition, to address students’ confusion regarding interdisciplinary content, introductory or preparatory knowledge modules can be offered at the beginning of the course to help students establish a basic interdisciplinary cognitive framework, supplemented by auxiliary learning resources.

In terms of the implementation path of the curriculum system, the CITM adopts a combination of modular teaching and project-driven teaching to provide students with an application platform of interdisciplinary knowledge through the practical scenarios of real projects. At the same time, emphasis is placed on the implementation of team collaboration and diversified evaluations to strengthen students’ practical abilities and teamwork skills. In the teaching implementation, teachers provide students with flexible combination of learning units through modular dismantling of curriculum content, and help them build a skillset tailored to their learning needs and future industry developments. In addition, through project-based teaching and interdisciplinary team collaboration, students are able to internalize theory and skills in design practice. The evaluation mechanism takes a variety of forms, including work reviews, process recording and project outcome evaluations, so as to comprehensively measure student learning outcomes and continuously optimize the curriculum content.

### 3.2. Innovations in teaching methods and approaches

In the construction of the CITM, the innovation of teaching methods and approaches is an important guarantee to improve teaching effect and promote the development of students’ comprehensive abilities [44]. Traditional teaching models often focus on the one-way transmission of knowledge, making it difficult to meet the demands of modern design education for practicality, interactivity and innovation. Therefore, this curriculum system introduces advanced teaching methods such as PBL [45], flipped classroom [46], and studio-based learning [47] during implementation, aiming to build a student-centered diversified teaching system. In PBL, students use real design projects as a platform to apply multidisciplinary knowledge in the problem-solving process, gradually cultivating their innovative thinking and teamwork skills. The flipped classroom breaks the limitations of traditional teaching in terms of time and space, encouraging students to independently study basic knowledge before class, and further consolidating and deepening their learning through group discussions and practical exercises during class. Studio-based teaching centers on creative practice. Under the guidance of teachers, students complete design tasks in simulated real-world working environments, thereby developing the ability to solve complex design problems.

In order to further improve the teaching effect, the curriculum system promotes in-depth interactions between industry, academia and students through interdisciplinary workshops. As a flexible teaching method, workshops can focus on specific issues in a short period of time and promote the rapid integration of interdisciplinary knowledge [48]. In the actual implementation, the teaching team invites industry experts from design, technology, business and other fields, as well as teachers from other disciplines to participate in the curriculum, through sharing experiences, guiding discussions and fostering teamwork, to provide students with multi-perspective approaches to problem solving. This format not only helps students understand the interconnections between various disciplines, but also enables them to be exposed to industry practices in advance to enhance professional literacy and adaptability. The diversity and flexibility of the interdisciplinary workshops provide students with valuable experience in interdisciplinary collaboration, while also providing an effective path for making the course content practical and applicable.

In addition, the use of digital teaching platform and online resources provides technical support for students’ self-directed learning and hands-on exploration. The curriculum system actively incorporates a variety of online learning resources and digital tools, such as online tutorials, virtual labs and knowledge sharing platforms, to create convenient learning channels for students. Teachers disseminate learning content, assignment tasks, and feedback suggestions through the teaching platform, enabling students to arrange their learning pace freely according to their needs and engage in interactive discussions with teachers and classmates via the platform.

### 3.3. Integration of interdisciplinary teaching teams and industry resources

The implementation of the CITM needs to be supported by high-level faculty and abundant industry resources, and creates conditions for interdisciplinary collaborative learning and practical application for students by integrating professional knowledge and practical experience from multiple fields both within and outside the institution. In terms of faculty development, the curriculum pays attention to the integration of multi-disciplines in the teacher team, and sets up an interdisciplinary teaching team composed of teachers from various fields such as design, art, computer, psychology and marketing. Through the collaborative teaching of teachers with multi-disciplinary backgrounds, the course can provide students with knowledge systems and ways of thinking from different disciplinary perspectives, helping them to stimulate creativity in the multidimensional knowledge intersection [49]. For example, design teachers can provide professional guidance in visual communication and creative design. Computer teachers help students master digital media technology and programming skills. Psychology teachers focus on teaching user behavior analysis and interaction design principles, while marketing teachers offer theoretical support in brand planning and marketing communication. This interdisciplinary teacher collaboration model not only reinforces the theoretical depth and technical breadth of the curriculum, but also facilitates knowledge sharing among teachers and the improvement of teaching ability, thus promoting the overall quality of the curriculum [50,51].

The curriculum actively cooperates with enterprises to further strengthen students’ practical skills and industry adaptability by introducing real cases and practical projects. School-enterprise cooperation is an important means of combining classroom teaching with industry demands, enabling students to gain insights into industry trends and workflows by participating in practical projects [52]. In terms of cooperation, enterprises can provide design requirements, data resources and case studies, while students work in teams to propose design solutions to specific problems and engage in field practice. For example, brand design courses can cooperate with advertising companies to carry out advertising planning and visual design around the needs of corporate brand upgrading. User experience design courses can partner with technology companies to participate in the development of user interface and interaction design for digital products. Through these collaborative projects, students are able to confront practical problems in design and complete solutions under the joint guidance of industry mentors and course teachers, so as to enhance their practical skills and professional competence, but also enables them to establish contacts with the industry during the school, and lay a solid foundation for future employment. In addition, the curriculum further enriches teaching content and practical opportunities by introducing industry mentors and establishing resource sharing platforms. As participants in the curriculum, industry mentors can provide students with insights and experiences from the forefront of the industry through special lectures, case analyses, and project evaluations. The resource sharing platform integrates the design needs of enterprises, industry cases and the latest technologies, making them readily available for students’ ongoing learning and reference.

### 3.4. Optimization of evaluation and feedback mechanism

In the implementation of the CITM, the optimization of curriculum evaluation and feedback mechanism is an important component to ensure teaching quality and promote the improvement of students’ comprehensive abilities [53]. The traditional evaluation methods tend to rely primarily on summative evaluation, emphasizing the evaluation of students’ learning outcomes, but ignoring the development and diversity of the learning process. Therefore, this curriculum proposes a diversified evaluation mechanism that comprehensively measures students’ learning outcomes and skill enhancements by combining both formative evaluation and summative evaluation, along with self-evaluation, peer evaluation and teacher evaluation in a collaborative approach. The formative evaluation focuses on the student’s performance during the learning process, such as their participation in class discussions, contributions to teamwork, and progress towards project completion. The summative evaluation emphasizes the quality of final learning outcomes, such as the completeness of design proposals, the expressiveness of portfolios, and the effectiveness of user feedback. By combining both, the evaluation mechanism can reflect students’ learning processes and outcomes more comprehensively, thereby promoting the sustainable development of their design skills and practical abilities.

In terms of the specific form of evaluation, the curriculum introduces multidimensional evaluation criteria, fully taking into account students’ learning experiences and the development of their professional competencies [54]. On the one hand, self-evaluation and peer evaluation, which reflect students’ self-reflection and teamwork ability, are key components of the evaluation mechanism. Through self- evaluation, students can summarize their own learning attitudes and achievements, thereby identifying their strengths and areas for improvement. Meanwhile, peer evaluation allows students to understand how their teammates perceive their performance, fostering a stronger sense of teamwork and enhancing collaborative skills. On the other hand, as the core component of the evaluation mechanism, teacher evaluation conducts in-depth analysis and guidance to students’ learning outcomes on the principle of professionalism and objectivity. It not only covers the creative and technical analysis of the design works, but also provides detailed feedback on students’ comprehensive performance in the course and provides directional suggestions for students’ subsequent learning.

In addition, the evaluation mechanism further strengthens its practice-oriented focus by primarily basing evaluations on portfolios, design proposals, market feedback and user testing data, thus comprehensively monitoring the enhancement of students’ overall capabilities. Portfolios, serving as an essential platform for demonstrating students’ design ability, act as a key tool for evaluating their creativity, design level, and aesthetic sensibilities [55]. Design proposals highlight students’ research skills and logical thinking in project work, reflecting their understanding of industry needs and their problem-solving ability. Market feedback and user testing data, as critical evaluation indicators in practice, collect user feedback on the use of design schemes from real environments, and directly reflect the practicality and market adaptability of students’ design works [56]. This evaluation mechanism not only underscores the course’s practicality and applicability, but also helps students clearly perceive their own progress and shortcomings, thereby stimulating their motivation and intrinsic drive to learn [57].

In summary, by establishing a multidimensional evaluation mechanism and optimizing feedback mechanism, the CITM can effectively enhance both the course’s teaching quality and students’ learning outcomes. The combination of formative evaluation and summative evaluation, along with self-evaluation, peer evaluation and teacher evaluation, makes the evaluation more comprehensive and scientific. The practice-oriented multidimensional evaluation standard further highlights the practicability and applicability of the curriculum. By dynamically tracking the improvement of students’ comprehensive abilities, the evaluation mechanism becomes an important tool for driving ongoing curriculum refinements, while also providing support for the innovative development of interdisciplinary design education model.

## 4. Case analysis

This study took the course module “Brand and User Experience Design” as an example, which aims to cultivate students’ abilities to comprehensively apply brand planning, user research and interaction design, and to carry out innovative design in combination with market demand under the background of digital communication. This course module covers multiple links such as user needs research, brand concept construction, user experience design and digital brand communication strategy, focusing on the combination and application of theory and practice. Students need to complete the entire design process from conceptual ideation to user testing to develop their multidimensional comprehensive design thinking and practical ability.

Design-based learning integrates design thinking and design process into the classroom, engaging students in real-world context. it is a natural approach and an essential part of a authentic STEAM program [14,58]. Therefore, the teaching practice of this study is conducted within an actual classroom context. First, the course module is designed to solve the difficulties that may be faced in the course implementation and improve its feasibility. Next, action research method is applied in the classroom to conduct interviews and collect data. Through participant observation, teachers gain deeper insights into the progress of the course, which is helpful for data analysis. Finally, triangulation increased the effectiveness of the study by conducting multi-directional cross-verification from different perspectives and positions [59], so as to obtain more objective and authentic conclusions.

### 4.1. Subjects

The recruitment period for the study was from July 16, 2024 to August 31, 2024. After the screening of the researchers, the final selection of a pilot class of course reform in the fall semester of the third-year majoring in VCD in a university in Nanjing. A total of 30 students and 5 teachers participated in the experiment, among whom the students were between 21 and 23 years old. Prior to this, the students had already received fundamental training in traditional VCD courses, possessing a solid foundation of knowledge and skills in VCD. However, their exposure to interdisciplinary content was relatively limited. The teaching team was composed of teachers with professional backgrounds in visual design, fine arts, computer technology, psychology and marketing. The corresponding teaching team of STEAM is shown in Table 1. In addition, on the basis of advocating and implementing the training program of thick foundation, wide caliber and specialization, this major adopts the teaching model of studio tutorial system, which sets up a research field for the reform and practice of the CITM. This provides a good experimental condition for this study, and also makes the implementation of the CITM more targeted and practical significance.

**Table 1:** The corresponding teaching team of STEAM in this study.

STEAM	Implement	Teachers	Professional backgrounds
Technology	Visual design tools and digital design technologies	A	Visual Design
Arts	Creative thinking and visual aesthetic expression	B	Fine arts
Engineering	Iterative processes, prototyping and testing stages of design projects	C	Computer Technology
Science	Application of user behavior research and cognitive psychology	D	Psychology
Mathematics	Application of data analysis, statistical methods or quantitative approaches in design evaluation	E	Marketing

The teaching implementation and classroom observation of this study were carried out in an intelligent classroom equipped with an independent recording and video studio. Before the study was conducted, we signed written informed consent for classroom observation with the subjects and have been approved by the author’s college review board (NUA SCI) for research on human ethics (approval number NUA SCI-E-2024–002). The researchers can observe students’ classroom learning from different angles through the computer screen, or directly observe from the glass window. The glass window is one-way viewing, and students in the classroom cannot see the situation in the studio.

### 4.2. The implementation process of teaching

The PBL was adopted in the teaching process, combining the characteristics of the flipped classroom and studio-based learning, and organizing students to carry out interdisciplinary collaborative design projects around real problems. The teaching was divided into the following stages:

(1) Project initiation and team formation: Students were grouped according to their personal interests and professional strengths to form 5 cooperation groups, each group was composed of 6 students, and they are respectively responsible for research, design, technical implementation, user testing and other tasks. A team of teachers explained the project objectives and workflow in detail during the start-up phase.
(2) Design survey and creative iteration: Students collected data through field research, user interviews and questionnaires, and then analyzed the survey results under the guidance of teachers to develop preliminary design concepts. Subsequently, each group formulated brand concepts and design directions, continually refining their design proposals through classroom discussion and teacher feedback.
(3) Interdisciplinary collaboration and project implementation: Each group worked according to its assigned tasks and drew on knowledge from multiple disciplines to complete the design projects. For example, “Brand concept formation and planning” carried out brand positioning, visual identity design and brand communication strategy planning under the guidance of the teachers specializing in visual design and marketing. “User experience design” involved collaboration under the guidance of art and computer technology teachers to jointly complete the design and implementation of the interactive interfaces.
(4) User testing and achievement exhibition: Students put their design works into the actual use scenario for user testing, gathering user feedback for final iterations and refinements. During the demonstration phase, industry experts and the teaching team were invited to provide a comprehensive evaluation informed by user testing data.

### 4.3. Action research

This study adopted the action research and took the teaching of the Brand and User Experience Design course as the research object to explore the effectiveness of the CITM of VCD based on the STEAM education. The action research emphasizes identifying and solving problems in actual situations, and promoting practical improvement through the interaction between researchers and participants [60]. This method is particularly suitable for educational innovation, as it facilitates a deep integration of theory and practice and allows for dynamic monitoring and continuous optimization of the implementation effectiveness of teaching model.

In this study, action research was divided into four stages: planning, action, observation and reflection. Drawing on the practical problems encountered by teachers from different professional backgrounds in their respective teaching processes, and incorporating the collected data, the researchers formulated a research plan, proposed corresponding solutions and approaches, and then implemented the plan and action measures. If it is found that the plan does not align with the actual conditions during the implementation of the teaching plan, it is revised to ensure the entire research process proceeds in an orderly manner and effectively solves the problems. During the action research process, the observation and record of the status of action prepared the foundation for follow-up research, and provided real and systematic materials for the results of action research, which is conducive to the comprehensive analysis of the results.

#### 4.3.1. Planning.

During the planning stage, the teaching team developed a detailed course implementation plan. Through the preliminary research and analysis of the current state of the course, the main existing problems were sorted out, including outdated teaching content, insufficient practical components and limited integration with industry resources. On this basis, the teaching team formulated specific teaching objectives, course content frameworks, implementation steps and evaluation criteria. The planning stage also included the collaborative arrangement of the teaching team and industry mentors, resource integration, student grouping strategies, and expected learning outcomes.

#### 4.3.2. Action.

During the action stage, the teaching team officially advanced the course teaching activities according to the plan. Relying on top-level design, teachers carried out teaching that combines theory with practice, organizing interdisciplinary co-teaching, special lectures and practical projects. Students, in accordance with the pre-determined groups, participated in the Brand and User Experience Design course tasks in groups, engaging the entire process practice from the initial research, brand planning to user experience design and the development of communication strategies. During the implementation of the action, teachers and industry mentors jointly guided students to complete tasks at each stage, adjusting teaching strategies as needed based on actual circumstances. Classroom activities included brainstorming, prototyping, stage reports, on-site feedback, etc., to enhance students’ teamwork and innovation abilities. Meanwhile, the researchers carried out data collection and process documentation at each key node to ensure the smooth progress of the action stage.

#### 4.3.3. Observation.

During the observation stage, the researchers conducted systematic and continuous observation and data collection throughout the teaching process. The specific methods included classroom participation observation, process documentation, interviews with teachers and students, questionnaires, and the evaluation of interim presentations. In addition to non-participatory observations conducted by the researchers, participatory observations could also be carried out by one or more entrusted students without disrupting classroom learning. By continuously documenting student group collaboration, the interaction between teachers and students, and project progress, the researchers collected learning logs, design sketches and interim assignments to comprehensively understand students’ learning dynamics and ability improvement. At the same time, students’ interim outcomes were evaluated by experts, enabling analysis of changes in their design thinking and innovative practice abilities. Through interviews and questionnaires, the feedback on course content and teaching methods was obtained from teachers and students.

#### 4.3.4. Reflection.

The researchers systematically sorted out and deeply analyzed the data and observation results collected in the previous stage. The teaching team held a reflection seminar to discuss aspects such as the achievement of course objectives, the improvement of students’ abilities, the organization and implementation of teaching, and the integration of resources. By summarizing the feedback from teachers and students, analyzing the performance of students’ works and the learning processes, the problems and shortcomings existing in course implementation were discovered, such as insufficient coherence between some content and uneven allocation of time for practical activities. Drawing on suggestions from industry mentors and experts, targeted optimization measures and improvement plans were proposed. Based on the reflections, researchers and teachers made timely adjustments to the course content and teaching processes, refined the CITM, and achieved a continuous cycle of improvement between theory and practice. The research team adhered to a spiral ascending process of “action – reflection – re-action” to continuously advance course reform and innovation.

### 4.4. Interviews

Prior to the formal interviews, preliminary interview questions were drawn up based on the content of the CITM, and pilot interviews were conducted with one teacher and one student. The results of these pilot interviews were then analyzed and the questions revised accordingly. The formal interviews included two parts. First, for the teacher interviews, after the course concluded, researchers conducted semi-structured interviews with the teaching team to discuss teaching content, teaching methods, evaluation and interdisciplinary teaching. Second, for the student interviews, students were interviewed during their spare time regarding learning effectiveness, co-teaching and ability enhancement. The interview outline is provided in S1 Appendix A and B.

#### 4.4.1. Interview coding and analysis for teachers.

This study conducted content analysis on semi-structured interview data from teachers with backgrounds in Visual design (A), Fine arts (B), Computer technology (C), Psychology (D) and Marketing (E), all of whom teach the Brand and User Experience Design course. Based on the interview content, fourteen core codes were extracted and summarized, covering four major thematic domains: teaching content (C), teaching methods (M), teaching evaluation and feedback (E) and Interdisciplinary teaching (I). The specific codes and definitions are shown in Table 2.

**Table 2:** Semi-structured interview coding for teachers.

Coding	Theme	Definition	Source
C1	Selection of teaching content	Teachers describe the composition and selection basis of the core content of the course, with an emphasis on the alignment between the content and the industry’s cutting-edge and demands.	A, C, E
C2	Stratified teaching design	Design differentiated teaching content or assignments according to students’ varying backgrounds and proficiency levels.	A, B
C3	Integration of theory and practice	Teachers emphasize the integration of theoretical content with practical application in the course.	A, B, C, D, E
M1	Diversity of teaching methods	Adopt various teaching methods, such as lectures, project-based teaching, flipped classroom, and studios, to enhance teaching effectiveness.	A, C, E
M2	Digital teaching application	Support teaching and assignment management by utilizing digital platforms and online tools.	A, C, D, E
M3	Advantages of offline teaching	Place emphasis on face-to-face teaching and on-site guidance, highlighting the importance of in-person interaction.	B
E1	Diversified evaluation mechanisms	Through the combination of formative evaluation and summative evaluation, as well as the collaborative approach of self-evaluation, peer evaluation and teacher evaluation.	A, B, C, D, E
E2	Practice-oriented evaluations	The course optimization and skill improvement are promoted through the evaluation of practical outcomes such as portfolios, design proposals, market feedback and user testing data.	A, B, C, D, E
E3	Insufficiency of soft skills evaluation	Insufficient attention is paid to soft skills such as team collaboration and emotional expression in the evaluation process.	A, B
E4	Difficulty in evaluating technical details	The detailed assessment criteria for technical skills are not well-developed, making quantification difficult.	C
I1	Advantages of interdisciplinary collaboration	Collaboration and complementary strengths among teachers from different disciplinary backgrounds enhance the depth and breadth of course design.	A, B, C, D, E
I2	Disciplinary divergence and resolution	Differences arise among teachers due to varying disciplines, and conflicts are resolved through consultation and data support approaches.	A, B, C, D, E
I3	Industry resources integration	Introduce resources such as enterprise projects, training bases, and industry mentors to enhance the integration of industry and education.	A, C, D, E
I4	Lack of enterprise practice	The connection between the course practice and enterprise and market resources is not deep enough.	E

In terms of teaching content, some teachers emphasized the industry relevance and cutting-edge of the course content (C1), pointing out that the content is closely aligned with trends in visual design, digital interaction technologies, and market demands. Additionally, some teachers addressed students’ varying levels of foundational knowledge through stratified teaching design (C2), providing learning content that range from basic to advanced difficulty. Moreover, the integration of theory and practice (C3) has become an important principle in course design, with teachers strengthening students’ applied abilities by incorporating practical cases and market research.

In terms of teaching methods, the integrated use of various approaches such as PBL and flipped classroom (M1), along with the support of digital platforms (M2), has significantly enhanced students’ engagement and interaction. Teachers from visual design, computer technology and marketing were more proactive in adopting digital teaching tools (M2), while fine arts teachers placed greater emphasis on in-person interactive teaching (M3), believing that face-to-face communication is more conducive to creative expression.

Diversified evaluation mechanisms (E1) and practice-oriented evaluations (E2) were widely implemented in the course. Through the combination of formative evaluation and summative evaluation, as well as the collaborative approach of self-evaluation, peer evaluation and teacher evaluation, and utilizing diverse evaluation bases such as portfolios, design proposals, market feedback and user testing data, the course provided a comprehensive reflection of students’ learning outcomes. However, teachers also pointed out shortcomings in the evaluation system regarding soft skills (E3) and evaluating technical details (E4), suggesting that the evaluation criteria should be improved by increasing the weight of team collaboration and emotional experience in the evaluation process.

Interdisciplinary collaboration (I1) was regarded as a key factor in enhancing the quality of course design and teaching. The complementarity of teachers from the five different professional backgrounds brought diverse perspectives to the course, fostering innovation in both content and methods. Disciplinary divergence (I2) was inevitable, but teachers achieved consensus through consultation and data support, ensuring effective team collaboration. Meanwhile, there was a strong demand for industry resource integration (I3). Teachers suggested deepening cooperation with enterprises, expanding training bases, and introducing industry mentors to address the current shortage of enterprise resources in the practical components of the course (I4).

#### 4.4.2. Interview coding and analysis for Students.

We selected 15 students who participated in the Brand and User Experience Design course for interviews. During the interview process, the researchers randomly selected one question from each of the four categories to interview the students, ensuring that all interview topics were covered. The statistics of student interview coding are shown in Table 3. According to the coding summary and qualitative analysis of this study, the CITM has shown significant effectiveness in enhancing students’ comprehensive abilities, optimizing the learning experience, acquiring new knowledge and enhancing skills. However, there are still certain challenges in aspects such as teaching content and evaluation mechanism.

**Table 3:** Statistics of interview coding for students.

Theme	Sub-theme	Frequency	Positive feedback	Negative feedback
Ability enhancement	Enhancement of interdisciplinary collaboration ability	9	Improved communication skills and collaboration awareness, with more reasonable division of labor	Conflicts and difficulties in coordination are common at the early stage of task allocation
	Enhancement of professional ability	8	Progress in areas such as brand planning, data analysis, user research, etc	The pressure of acquiring new knowledge is high, and some students lack sufficient foundational skills
	Enhancement of innovative thinking ability	7	Think more broadly and learn to view problems from multiple perspectives	Limited time available for innovative practice
	Enhancement of practical ability	9	Significant gains from integrating theory with practice	Short project duration and tight schedule for implementation
Teaching model	Co-teaching by multiple teachers	13	Interdisciplinary complementarity and rich content	Content overlap and lack of continuity among teachers
	Diversified teaching content	12	Learning from multiple perspectives with significant gains	Content is scattered and lacks systematicness
	Integration of project-based practice with classroom learning	11	Highly practical and closely aligned with real-world work	Integration of theoretical instruction and practical application could be further improved
	Time allocation/ pacing	9	--	Fast pace and tight schedule
Knowledge and skills	Acquisition of new knowledge/ skills improvement	10	Acquired new knowledge such as research, branding, and user experience	Too much content and insufficient connection between some knowledge areas
	Teamwork and communication	11	Learned to listen, express opinions, and resolve disagreements	Communication barriers and conflicts of ideas
	Challenges and coping strategies	8	Solved problems through communication, division of labor, and feedback mechanisms	Heavy workload and uneven team efficiency
	Evaluation feedback	5	Comprehensive evaluation with multi-dimensional evaluation	High subjectivity and peer evaluation may lack fairness
Attitudes and Suggestions	Overall satisfaction	13	Satisfaction/ Support	Some students hope to optimize the process and evaluation
	Impact of the course on employment	7	Practical and interdisciplinary experiences enhance employability	--
	Changes in learning motivation	6	Increased motivation and initiative	--
	Suggestions for improvement	12	--	Students hope to unify the progress, optimize tasks, and strengthen discussions

Firstly, students generally report that they have gained improvements in various abilities through the course. Most interviewees believed that interdisciplinary cooperation and team communication skills have significantly improved, enabling them to better coordinate respective divisions of labor and cooperation in actual projects. In addition, professional knowledge and skills such as brand planning, user research and data analysis have also been systematically trained and applied. It is worth noting that students have achieved particularly remarkable gains in innovative thinking and project practice. Interdisciplinary brainstorming and hands-on experience with real projects have greatly stimulated their creative thinking and practical operation abilities. This finding is consistent with the viewpoint in the existing literature that PBL can effectively promote the development of innovation ability [61,62].

Secondly, the CITM has received positive feedback from the majority of students. The interviewed students believed that the co-teaching by teachers from different professional backgrounds not only greatly enriches the course content but also broadens their cognitive horizons, helping them fully understand the entire process of brand and user experience design. However, some students pointed out that insufficient coordination among teachers sometimes led to content repetition and disruptions in the course flow, which affected the learning experience for some. This finding suggests that in the process of co-teaching by multiple teachers, there is a need to further strengthen communication among teachers and the integration of teaching content to enhance the coherence and systematicness of the course.

Thirdly, teamwork and division of labor have become key aspects in the students’ learning process. Some students encountered communication barriers or even disagreements in the early stages of collaboration due to differences in professional perspectives and ways of thinking. However, most teams were ultimately able to achieve effective collaboration through active communication and clear division of tasks. Students felt that this process has exercised their teamwork and problem-solving abilities, which are highly relevant for their future careers.

Finally, regarding the course evaluation mechanism, students agreed that the diversified evaluation model provides a more comprehensive reflection of both individual and team learning outcomes. However, some students pointed out that there is a certain degree of subjectivity in peer evaluation and formative evaluation, making it difficult to fairly reflect the actual contributions of some team members and occasionally resulting in “free-riding” situations. Therefore, further refining and detailing the evaluation criteria, as well as strengthening formative evaluation and feedback, are key to enhancing the fairness and incentive mechanisms of the course.

Overall, the vast majority of interviewed students expressed strong approval of the CITM, believing that it effectively enhanced their professional competence, innovative ability and employability, while also stimulating their motivation to learn and team spirit. However, challenges remain in the implementation of the course, such as teacher collaboration, course pacing and task allocation, and the evaluation mechanism. In response, it is recommended that future course development further optimize the teaching organization structure, strengthen the coordination among teachers and the integration of content, scientifically arrange the project schedules, and improve the diversified evaluation mechanism, so as to continuously enhance the teaching quality of interdisciplinary courses.

### 4.5. Questionnaire

#### 4.5.1. Questionnaire design.

To investigate the learning experience and perceptions of the CITM, this study designed a learning experience questionnaire for the Brand and User Experience Design course. First, we formed a ten-member expert panel to discuss the questionnaire items, with the panel consisting of both teachers and students. Among them, five teachers were from the teaching team of this course, and the other five were students majoring in design who had not participated in the course. After multiple rounds of discussion and review by experts, a scale that is more targeted and scientific was finally determined. A 7-point scale was used for all items ranging from “strongly disagree” (1) to “strongly agree” (7). To ensure that the survey items were appropriate for the context of this study, we conducted a pre-test on the scale. The pre-test helped us identify ambiguous expressions, clarify the wording of the items, and further refine the questionnaire so that respondents could fully understand the content and the quality of the survey could be improved. In the end, the questionnaire included 5 dimensions, each with 4 items, for a total of 20 items. Table 4 shows the complete questionnaire items. We administered the questionnaire to 30 students who participated in the course, and a total of 30 completed questionnaires were collected.

**Table 4:** Learning experience questionnaire for the Brand and User Experience Design course.

Constructs	Question number	Questionnaire items
Course content and Cognition	Q1	The content of this course helps me understand the core concepts of brand and user experience design, as well as their practical applications.
	Q2	The theoretical knowledge in the course is closely integrated with practical projects, which has increased my interest in learning.
	Q3	I am able to understand the interdisciplinary knowledge of brand planning, user research, and interaction design covered in the course.
	Q4	Through this course, I have gained a clearer understanding of interdisciplinary integrated design.
Project-based learning and practical operation	Q5	The project-based learning and real case-based learning have enhanced my enthusiasm for learning.
	Q6	The division of labor and cooperation in the project team enabled me to better exert my professional expertise
	Q7	By participating in the project, my creative thinking has been exercised and improved.
	Q8	The arrangement for aspects such as user needs research, brand concept construction, and user experience design in the projects is reasonable.
Interdisciplinary collaboration experience	Q9	Members can communicate effectively with each other and integrate multidisciplinary knowledge during group collaboration.
	Q10	Collaborating with classmates from different professional backgrounds is clearly beneficial to me in the project.
	Q11	The multidisciplinary guidance from the teaching team has promoted my understanding of the integration of design and technology.
	Q12	During the implementation of the project, I took the initiative to learn and apply relevant knowledge from other disciplines.
Personal growth and ability enhancement	Q13	This course has enhanced my ability to analyze and solve practical problems.
	Q14	I have gained a deeper understanding of knowledge related to brand planning, user experience design and digital communication strategies.
	Q15	The user testing and expert review stages helped me recognize the shortcomings of my design solutions and make improvements.
	Q16	After completing the course, I feel more confident in participating in interdisciplinary design projects.
Overall evaluation of the course	Q17	The reasonable structure and rich content of the course can stimulate my motivation to learn.
	Q18	The cross-integrated teaching model suits my learning style.
	Q19	Through this course, I have gained new perspectives on my future career development.
	Q20	I am willing to recommend this course and the CITM to other students.

#### 4.5.2. Analysis.

This study used SPSS 27 to conduct data analysis. The reliability test value of Cronbach’s Alpha for the 20 items of the scale was 0.911, indicating that the scale has good internal consistency. The validity was verified using the KMO and Bartlett’s tests. The KMO value was 0.732, and the Bartlett’s test of sphericity reached a significant level (p < 0.001), indicating that the scale has good validity.

**Fig 2:**
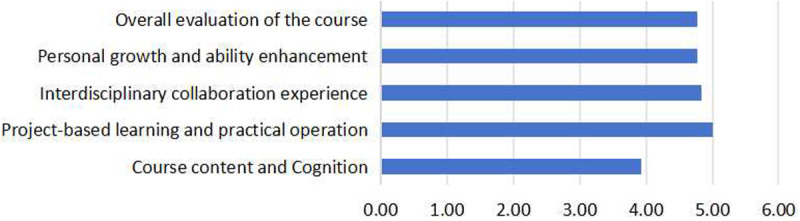
Comparison of the average scores on the dimensions in the questionnaire for students.

The comparison of the average scores for the 5 dimensions in this questionnaire is shown in Fig 2. The results indicated that students have the highest level of recognition for PBL and practical operation (M = 5.00), suggesting a strong preference for enhancing their personal abilities and skills through practical experience and actual projects, fully demonstrating the advantages of practical teaching in interdisciplinary courses. In contrast, the dimension of course content and cognition received the lowest score (M = 3.93), reflecting significant difficulties students face in understanding theoretical knowledge and interdisciplinary content. This indicates that the course design and teaching methods in this aspect require further optimization. The scores for interdisciplinary collaboration experience (M = 4.84), personal growth and ability enhancement (M = 4.78), and overall evaluation of the course (M = 4.77) were similar, all falling within a moderately high and positive range. This suggests that both interdisciplinary collaboration and personal development are consistently recognized and valued within the course, although there remains room for further improvement. In summary, the course excels in practical teaching and project-based activities, but the theoretical and cognitive aspects of instruction still need to be strengthened and improved.

**Fig 3:**
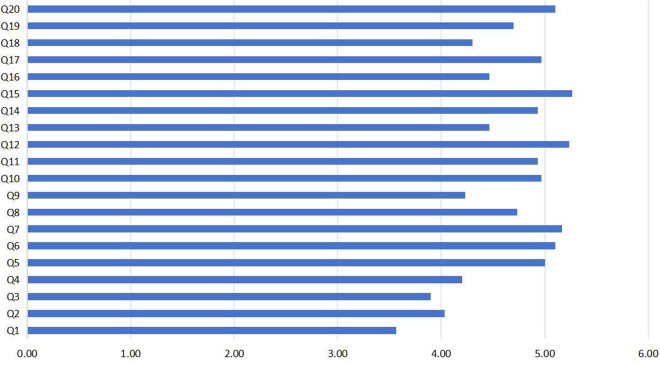
Comparison of the average scores on the items in the questionnaire for students.

Additionally, the comparison of the average scores for each item in this questionnaire is shown in Fig 3. The results showed that the two aspects with the highest scores are the contribution of user testing and expert review to improving design solutions (Q15, M = 5.27), and students’ initiative in learning and applying knowledge from other disciplines (Q12, M = 5.23), indicating that students highly recognize the importance of practical feedback and interdisciplinary knowledge integration in their learning process. Furthermore, the score for the exercise and improvement of creative thinking (Q7, M = 5.17) was also relatively high, indicating that the course effectively fosters students’ creative thinking abilities. In contrast, lower scores were observed for the extent to which the course content helps students understand core concepts of brand and user experience design (Q1, M = 3.57), and for the depth of understanding of interdisciplinary knowledge (Q3, M = 3.90), reflecting students’ difficulties and shortcomings in grasping theoretical knowledge and achieving comprehensive interdisciplinary cognition. Overall, the analysis suggests that while students generally recognize the value of project-based practice and interdisciplinary collaboration, there remains room for further improvement and optimization in terms of theoretical depth and systematic integration of interdisciplinary knowledge within the course design.

### 4.6. Triangulation

**Fig 4:**
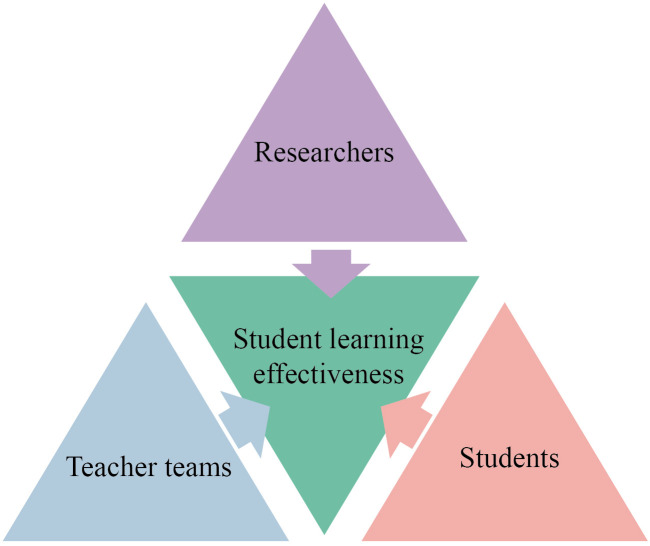
Structure of the triangulation.

Based on the triangulation, as shown in Fig 4, this study deeply analyzed the effects and problems in the implementation of the Brand and User Experience Design course from three different perspectives: researchers, the teaching team, and students, in order to understand the overall teaching quality of the course more objectively.

From the perspective of the researchers, they systematically observed and recorded the entire process of course implementation through the action research, which involves four stages: planning, action, observation and reflection. The researchers pointed out that interdisciplinary collaboration and project-based teaching method have effectively enhanced the interactivity and practicality of teaching, significantly improving students’ comprehensive design thinking and practical abilities. Furthermore, they also emphasized that data obtained through interviews and questionnaires indicated that students made notable progress in teamwork, interdisciplinary integration, and hands-on practice. However, it was also observed that insufficient communication within the teaching team has led to discontinuities in the teaching pace, and that the evaluation mechanism has certain shortcomings in evaluating individual contributions within the team.

From the perspective of the teaching team, the teachers highly recognized the positive impact of the CITM on enriching teaching content and enhancing practical application. Teachers from various disciplines effectively increased the adaptability and practicality of the course through stratified teaching design and innovative teaching methods. However, the teachers also pointed out some problems. Although the CITM has been well received and has effectively promoted the diversity in course content, teachers focused more on addressing the challenges of collaboration caused by differences in professional perspectives during implementation. According to interviews with teachers, those from different disciplinary backgrounds have cognitive differences regarding course arrangement, teaching method selection, and the integration of industry resources, which leads to a lack of coordination in the connection of teaching content. Furthermore, they suggested that more active cooperation with industry should be carried out to enhance the authenticity and industry relevance of practical teaching components.

From the students’ perspective, those who participated in interviews and questionnaire believed that the CITM has significantly enhanced their innovation abilities and comprehensive skills, especially in terms of interdisciplinary collaboration and hands-on project experience. However, students also pointed out several issues. For instance, the communication barriers within the cooperation groups were prominent in the early stage, particularly when the incoherence of the teaching content and pace negatively affected their learning experience. Students hope that the teaching team can strengthen internal communication, clearly define the teaching content and time allocation for each stage, and establish a more detailed and fair process-based evaluation mechanism.

## 5. Discussion

### 5.1. Research findings

The results of this study indicated that the CITM significantly enhance students’ innovative practical skills, communication abilities and teamwork awareness. These findings are consistent with the conclusions of Allina and Harris and de Bruin, who found that STEAM education promotes creativity [63,64]. It also supports the recent arguments by Xu, who highlighted the advantages of innovative interdisciplinary models in improving students’ interdisciplinary communication and teamwork [65]. Furthermore, the diverse teaching methods adopted in the course, such as PBL, flipped classrooms and workshops, have further enhanced students’ adaptability to tackle complex design tasks. It is consistent with Kokotsaki et al., who discussed the effectiveness of PBL in stimulating students’ comprehensive abilities in higher education [45].

During the process of action research, this study implemented the course following a cyclical structure of “planning–action–observation–reflection”, and employed triangulation for cross-validation, which effectively enhanced the objectivity and reliability of the research. This process echoes the theoretical discussions by Gibbs et al. regarding the potential of action research to drive educational innovation in higher education [60]. The interviews and questionnaires with students indicated high evaluations of the course’s practical orientation and diverse teaching methods, but relatively lower scores in their understanding of interdisciplinary theoretical content. These results highlighted the strengths of STEAM in promoting practical abilities, while also revealing shortcomings in the support for theoretical knowledge acquisition.

In addition, an unexpected finding during the course implementation was that, although some students initially expressed confusion about the CITM, their learning enthusiasm and initiative increased significantly in the later stages of the course, demonstrating an active willingness to explore complex problems. This suggests that students may require a longer period for cognitive adaptation, and that more scaffolding support should be provided in the early stages of instruction. This insight offers important guidance for the design of future courses.

### 5.2. Curriculum optimization

Following the characteristics of the VCD and considering practical teaching conditions, the proportion of practical teaching is increased in the curriculum. A scientific and practical teaching plan is developed to enhance hands-on training within the courses. Compared to theoretical teaching, practical teaching is more interdisciplinary, cross-domain and multidimensional, and its outcomes demonstrate greater originality and social applicability [66]. To help students with different proficiency levels better adapt to the CITM, teachers can employ a progressive scaffolding teaching approach that gradually advances from basic theories to more complex practices, while also providing a variety of learning resources and individualized guidance. In terms of practical teaching methods, the teaching objectives and tasks are accomplished through the joint participation of both teachers and students. Teachers provide students with favorable practical conditions and offer detailed guidance. Therefore, students should be the main focus, given sufficient time for reflection, and encouraged to develop problem-solving skills, enabling them to assess their learning outcomes through practical activities.

Horizontally, it pays attention to the system and intersection of the curriculum system, as well as the flexibility of the teaching content and format. In the past, the period allocation of professional courses adopted a phased and modular setting. This traditional setting leads to more concentrated teaching periods, while students have different ability to accept new knowledge, which is not conducive to teaching according to their aptitude. In the CITM, the overall structure and direction of the curriculum are determined through the discussion of the teaching team meeting, and the specific teaching content is flexibly mastered and designed by teachers with different professional backgrounds. The prerequisite is that the specific teaching content needs to be presented to the teaching team during meetings to achieve the cross-integration of different majors and the heterogeneous complementarity in team collaboration. For example, in the form of “One course, many lecturers”, some design courses can be jointly planned by multiple teachers, who collaboratively develop the syllabus and teaching plan. The key points of teaching are discussed and determined through the teaching team meetings. According to the course content, the teachers whose research areas align with the topic are selected to teach, and these teachers typically have complementary advantages in their respective research fields.

Vertically, an organic and open spiral curriculum system is constructed. A spiral curriculum refers to the repetition of course content at different stages while gradually expanding the scope of knowledge and deepening the level of specialization, forming a spiraling upward structure [67]. In the CITM of VCD, the courses taught by teachers from different professional backgrounds can be regarded as individual strands within a spiral structure, which together form several interwoven spiral curriculum systems [68]. Each spiral structure has undergone the process from design theory to design practice. With the advancement of the course, these spiral structures cross and integrate with each other, enabling students to continuously deepen and expand their professional knowledge [69]. The organic nature of the curriculum system implies that there are symbiotic and complementary relations between different areas of professional knowledge, while its openness refers to the constant communication and interaction between them [70]. Therefore, this organic and open spiral curriculum system allows knowledge from different disciplines to alternate within relevant courses, achieving symbiosis and complementarity, exchange and interaction, thereby further deepening and reinforcing the acquired knowledge.

### 5.3. Teacher collaboration and disciplinary integration

The diversification of design requirements is reflected not only at the professional level but also in the diverse learning experiences of teachers and the diversity of faculty structures. In the process of action research, the most valuable resource is teachers’ ability for self-improvement. Teachers should not only master their own professional knowledge but also actively engage in learning and practicing various extended disciplines, such as visiting engineer programs and school-enterprise collaborations. This helps them develop a multidisciplinary knowledge framework, provide students with a diverse learning environment, and enhance the implementation of the interdisciplinary teaching with greater diversity.

In team teaching within the CITM, it is emphasized that teachers with different professional backgrounds make full use of their professional strengths through mutual cooperation and heterogeneous complementation, so as to better accomplish the teaching objectives together. However, the study has found that each teacher in the team expresses different opinions or perspectives on the design and teaching methods of VCD courses in each teaching session discussion. Each teacher demonstrates a strong sense of professional loyalty to their own discipline, or a professional complex*.* Gosling argued that teachers have undergone systematic training in a specific discipline and are accustomed to developing courses within their own field. This deep-seated barrier stems from the existing “academic tribes or territories,” where the pressure of team teaching arises from the necessity for teachers to step out of their “comfort zone,” which is their own area of expertise [71]. Therefore, in order to achieve good collaboration and integration within the teacher team and effectively carry out team teaching, the teachers in the team must solve the “conflict” among the majors, enhance their sense of teamwork, pay attention to the coordination of the curriculum, and improve the integration of professional knowledge, so as to create a multidisciplinary and diversified teaching situation.

### 5.4. Students’ learning outcomes and course experience

In the learning process, students can easily grasp the content of VCD curriculum by receiving knowledge from different design directions. At the same time, this approach also presents certain challenges to their independent learning ability. From the students’ perspective, the degree of the cross-integration of curriculum depends on the teamwork of teachers. This model is more effective in stimulating students’ learning interest and participation more than the previous teaching model, and fosters their multidimensional creative thinking and comprehensive innovation ability. According to the results of the interview survey, students’ high evaluation of the CITM mainly stemmed from the effective integration of knowledge from different disciplines. The key factor behind the positive feedback on this new teaching model is the teachers’ ability to successfully integrate knowledge and perspectives from various fields in a cohesive manner. Furthermore, during the action research process, it was found that students show greater interest in hands-on practice than in theoretical explanation. Therefore, in theoretical courses, teachers can simplify textual content as much as possible by enriching the course content with actual case analyses or using engaging and life-oriented image materials. And, presenting theoretical knowledge in a visualized format can enhance students’ interest in theoretical learning, making it easier for them to understand the information conveyed by the teachers.

## 6. Conclusions

This study centered on the transformational needs of VCD education in the context of digitalization and interdisciplinary integration. Based on the STEAM education, it constructed the CITM for the field of VCD from four aspects: top-level design of curriculum, innovations in teaching methods and approaches, integration of interdisciplinary teaching teams and industry resources, and optimization of evaluation and feedback mechanism. This study adopted the action research, taking the Brand and User Experience Design course as a case example. Data were collected through classroom observation, interviews, questionnaires and team discussions, and multi-dimensional qualitative analysis was conducted using triangulation. The results indicated that the teaching model effectively enhances students’ innovative thinking, interdisciplinary collaboration and practical abilities, while also improving the alignment between the course and actual industry needs. Measures such as PBL, interdisciplinary teacher collaboration and the integration of industry resources have significantly promoted the development of students’ comprehensive quality and teamwork ability. Both students and teachers highly recognized the positive changes brought about by the CITM. However, challenges remain in students’ understanding of interdisciplinary theory and in teacher team collaboration. It is necessary to further improve the scaffolding teaching strategy, optimize mechanisms for teacher communication and collaboration, and strengthen the coherence of course content as well as the continuous integration of industry resources.

## 7. Limitations and future research

Although this study has achieved significant results in theoretical exploration and practical application, there are still some limitations that need to be further addressed. First, the efficiency of the integration of teaching resources and the management of the complexity of the curriculum system still need to be improved, which puts forward higher demands for the sustainability of educational practice. Second, the adaptability of some students in interdisciplinary education is still insufficient, which affects the universality and diversity of teaching outcomes to a certain extent. Third, based on a respect for the knowledge boundaries and disciplinary domains of each field, how to optimize the organizational structure of the VCD and appropriately address the issue of “disciplinary boundaries” within the new curriculum also requires further in-depth exploration.

Therefore, future research may focus on the following directions: First, improving the operability and flexibility of the curriculum and further optimizing teaching implementation approaches to better adapt to the complex demands of interdisciplinary education. Second, improving the scaffolding teaching strategy to strengthen the cultivation of students’ interdisciplinary adaptability. Third, investigating optimization schemes for the organizational structure of VCD to effectively address the issue of “disciplinary boundaries” in the interdisciplinary context. Fourth, exploring the application of emerging technologies, such as artificial intelligence and VR, in the innovative practice of interdisciplinary education, with the aim of building a more universal and forward-looking interdisciplinary teaching model.

Finally, it is worth noting that this study was conducted in the context of Chinese university, which may limit the universality of the conclusion. Future research could explore ways to more effectively promote the CITM in different countries or educational systems by adapting course content across cultures, establishing flexible interdisciplinary collaboration mechanisms, and optimizing localized teaching support systems.

## Supporting information

S1 appendix Appendix A & B. (DOCX)

S1 Original data Questionnaire - Original data. (XLSX)
