# Supplementary material for: Reform and practice of the cross-integration teaching model: A case study of visual communication design
Source: PLoS One. 2025 Jul 11;20(7):e0327813. doi: 10.1371/journal.pone.0327813 (PMC12250693; doi:10.1371/journal.pone.0327813)
Supplement: S1 appendix — (DOCX) [file pone.0327813.s001.docx]

**Appendix A**

**Interview outline for teachers. (During the interview, further questioning and discussion could be conducted according to the teacher’s answers.)**

| **Teaching content**  1. What kind of the most important teaching content are included in the courses?  How are these course contents selected?  2. Do you think these teaching content meet students’ learning needs?  Were there contents designed for students with different levels?  3. How do you ensure that the teaching content are cutting-edge and practical?  Were the latest industry developments incorporated to enhance the industry suitability of the courses? |
| --- |
| **Teaching methods**  1. What teaching methods do you usually use to convey knowledge to students?  How do students respond to these teaching methods?  2. How do you choose the appropriate teaching methods according to the course contents?  Can you give some examples of specific applications?  3. In the teaching process, what is the effect of PBL, flipped classroom or studio?  Do you use digital teaching platforms and online resources? |
| **Teaching evaluation and feedback**  1. Do you think that the diversified evaluation mechanism precisely reflects the learning process and learning objectives?  Are there any parts of the evaluation criteria that needs to be strengthened or adjusted?  2. Do you think that the introduction of practice-oriented (such as portfolios, design proposals, market feedback, user test data, etc.) evaluation mechanism can effectively promote the dynamic optimization of the course?  Do these effectively promote students’ comprehensive ability in complex problem solving?  3. What do you think of the effectiveness regarding the course evaluation mechanism that comprehensively assesses students’ learning outcomes?  Are any disadvantages that need to be eliminated in the implementation process? Would you please provide some specific examples to illustrate? |
| **Interdisciplinary teaching**  1. In the CITM, how do you view the collaboration and complement between teachers from different subject backgrounds?  How do you think this collaboration has influenced course design and the development of teaching methods?  2. Did you encounter any disagreements in the co-teaching due to differences in professional expertise?  If so, how did you handle and resolve these conflicts to promote effective collaboration within the team?  3. How do you think interdisciplinary teaching and the integration of industry resources can improve the overall quality of course?  In terms of the integration of faculties and industry resources, what improvements would be made? |

**Appendix B**

**Interview outline for students**

| **Student learning effectiveness**  1. How do you think that your professional ability has improved through the CITM?  2. What knowledge or skills have been most helpful for you during the learning process?  3. What challenges did you face during the process of your study and how to overcome them?  4. Which aspects of the CITM will best promote your learning effectiveness?  5. Do you benefit tremendously from the co-teaching conducted by faculty members with different disciplinary backgrounds? |
| --- |
| **Co-teaching**  1. What do you think of the co-teaching model in the course?  2. Do you need faculty members with different discipline backgrounds to teach you？  3. How does the co-teaching model affect your learning experience?  4. How do you think that the co-teaching has improved your understanding of the course contents?  5. How do the teaching styles of different teachers affect your learning in the co-teaching? |
| **Teaching in the cross-integration course**  1. How does the CITM affect your ability to think creatively?  2. What kind of achievement you obtain most from the cross-integration course?  3. Compared with the traditional teaching model, what advantages do you think the CITM has?  4. How do you think learning knowledge in different majors has helped you?  5. Do you think that the diversified evaluation mechanism is reasonable? |
| **Students’ general attitudes**  1. In general, what is your attitude toward the CITM?  2. Does the CITM meet your expectation?  3. Do you think the CITM is helpful for your future employment?  4. Has your learning motivation changed through getting engaged in the CITM?  5. What do you think needs to be improved about the CITM? |
